# A potential alternative to fungicides using actives-free (meth)acrylate polymers for protection of wheat crops from fungal attachment and infection†

**DOI:** 10.1039/d3gc01911j

**Published:** 2023-08-17

**Authors:** Liam A. Crawford, Valentina Cuzzucoli Crucitti, Amy Stimpson, Chloe Morgan, Jonathan Blake, Ricky D. Wildman, Andrew L. Hook, Morgan R. Alexander, Derek J. Irvine, Simon V. Avery

**Affiliations:** a School of Life Sciences, University Park, University of, Nottingham Nottingham NG7 2RD UK simon.avery@nottingham.ac.uk; b Centre for Additive Manufacturing, Department of Chemical and Environmental Engineering, University Park, University of Nottingham Nottingham NG7 2RD UK derek.irvine@nottingham.ac.uk; c Advanced Materials and Healthcare Technologies, School of Pharmacy, University Park, University of Nottingham Nottingham NG7 2RD UK; d RSK ADAS Ltd, Rosemaund, Preston Wynne Hereford HR1 3PG UK

## Abstract

Fungicidal compounds are actives widely used for crop protection from fungal infection, but they can also kill beneficial organisms, enter the food chain and promote resistant pathogen strains from overuse. Here we report the first field crop trial of homopolymer materials that prevent fungal attachment, showing successful crop protection *via* an actives-free approach. In the trial, formulations containing two candidate polymers were applied to young wheat plants that were subject to natural infection with the wheat pathogen *Zymoseptoria tritici*. A formulation containing one of the candidate polymers, poly(di(ethylene glycol) ethyl ether acrylate) (abbreviated DEGEEA), produced a significant reduction (26%) in infection of the crop by *Z. tritici*, delivering protection against fungal infection that compared favourably with three different commercially established fungicide programmes tested in parallel. Furthermore, the sprayed polymers did not negatively affect wheat growth. The two lead polymer candidates were initially identified by bio-performance testing using *in vitro* microplate- and leaf-based assays and were taken forward successfully into a programme to optimize and scale-up their synthesis and compound them into a spray formulation. Therefore, the positive field trial outcome has also established the validity of the smaller-scale, laboratory-based bioassay data and scale-up methodologies used. Because fungal attachment to plant surfaces is a first step in many crop infections, this non-eluting polymer: (i) now offers significant potential to deliver protection against fungal attack, while (ii) addressing the fourth and aligning with the eleventh principles of green chemistry by using chemical products designed to preserve efficacy of function while reducing toxicity. A future focus should be to develop the material properties for this and other applications including other fungal pathogens.

## Abstract 2

Fungicide-free (meth)acrylate polymer sprays help protect wheat crops from fungal infection.

## Graphical abstract

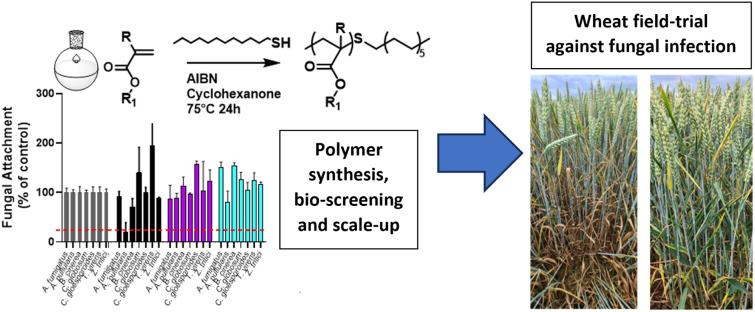

## Introduction

Fungi play crucial roles in multiple ecological processes and are an important source of food and pharmaceuticals.^1,2^ However, fungal colonisation can also lead to human and crop disease, material degradation and food spoilage.^3–6^ Fungicides are existing control treatments against fungal pathogens of crops, but these actives are undermined by growing pathogen resistance, substantial environmental concerns (including killing of beneficial fungi) and regulatory restrictions.^4^ Therefore, there is a strong incentive to develop effective and greener methods to prevent colonisation of problematic fungi in agricultural environments. This has added importance because fungi and fungus-like organisms destroy crops each year that would be sufficient to feed approximately 600 million people.^7^ Wheat is one of the most economically and nutritionally important cereal crops but 5–10% of yields are lost to infection by the fungus *Zymoseptoria tritici* even with the use of resistant crop varieties and fungicides.^8^

Fungal colonisation on surfaces is a first key step in many infection processes and is typically a three-stage process. The first stage is weak attachment of a few fungal cells/spores on a surface *via* pre-formed adhesins, such as hydrophobins.^10,11^ Irreversible attachment then occurs *via* spore germination and/or production of specialised adhesins and the secretion of extracellular material.^11,12^ The third stage, proliferation, increases the fungal burden, spore production is initiated, extracellular matrix synthesis increases, and the secretion of digestive enzymes occurs. It is in the proliferation stage where most of the degradation or infection occurs.^12–14^

Current methods used in agriculture to mitigate the infection process typically rely on the development of disease-resistant cultivars, and/or repeated application of fungicides, the latter particularly during leaf emergence.^5,8,9^ On more robust biological surfaces, fungal eradication can rely on more reactive chemistries, including heavy metal ions. For example, in wood-based materials copper-based chemistries are principal methods of preservation, and also in crop protection in some countries, but such actives are subject to tightened regulations in many countries. Moreover, active agents like these select for fungicide resistance among the fungi, eroding their efficacies.^9,15^

One alternative approach to reducing fungal colonisation generally is to prevent the initial attachment stage. This has been attempted using active agents (such as metal ions or fungicides) embedded in/or on a suitable matrix, with the agents often released over time and/or in response to environmental changes.^16,17^ Adopting such an approach helps ensure continued exposure of the fungi to the actives, which may either prevent spore germination or be fungicidal upon contact. This strategy also potentially reduces the need for multiple applications, but the use of active inhibitors in such approaches still raises the environmental concerns mentioned above. Spraying actives to crops can present particular environmental issues, where the active becomes more widely dispersed than just in the targeted area.

By contrast to above, the present study proposes that the prevention of fungal colonisation could be achieved using an actives-free approach, through non-eluting polymer coatings that only influence surface characteristics, *e.g.*, of leaves. This strategy is based on previous work that has focussed primarily on control of colonisation by human pathogens through the use of different surface chemical properties of polymers for treatment of medical device surfaces.^18–20^ The lack of an active antimicrobial in this type of approach also helps alleviate certain potential barriers to regulatory approval and commercialisation, while reducing the selection pressure on the target organism(s) to evolve resistance. This strategy also significantly alleviates the issues noted above concerning spray application, as the spraying mixture would comprise a large molecular weight, polymer exempt material. Thus, mitigating environmental impacts of treatment. Related polymers have a record of safe application in the field as fungicide-formulation adjuvants, *e.g.*, methyl-methacrylates for facilitating active fungicide delivery in the Atlox™ 4913 adjuvant. Adapting the actives-free anti-attachment approach to the agricultural sector could be particularly valuable as fungicide treatments contribute significantly to the cost of food production and the ecological impacts of farming.^15,21^ Accordingly, it would also help address the fourth principle of green chemistry through use of chemical products designed to preserve efficacy of function while reducing toxicity. Additionally, while not a monitoring technology, it links into the eleventh principle by reducing the potential for release of hazardous agrochemical actives into the biosphere.

The recent work that led to the hypothesis for this study adopted a high-throughput homopolymer screening approach to identify specific (meth)acrylate polymers that passively resist attachment of bacteria, or yeast cells of *Candida albicans* and spores of the filamentous fungus *Botrytis cinerea* (human- and crop-pathogenic fungi, respectively), corroborating the potential of an actives-free approach.^19,20,22,23^ The work with the fungi (subject of European and US patent applications 21707379.0 and 17/799914, respectively), also showed no toxicity-related growth inhibition when these species were contacted with the polymers while further laboratory assays indicated the effectiveness of certain polymers in protecting leaf samples from infection by *B. cinerea*, with no evidence of phytotoxicity.^20^ In the present study, we sought to exemplify the validity of the actives-free strategy by optimising the polymerisation procedures for key homopolymer candidates identified in the previous study, up-scaling the production to the kilogramme level and then applying the resultant materials to wheat plants in a natural-infection field trial. Such testing up to the commercial farm scale is a crucial step for validating the key proposed agricultural application of this protection strategy, that depends on surface characteristics alone. Consequently, the most promising polymers were those taken forward to the wheat field trial and subjected to natural infection by *Z. tritici*.

## Materials and methods

### Materials

Diethylene glycol ethyl ether acrylate (DEGEEA), di(ethylene glycol) methyl ether methacrylate (DEGMA), mono-2-(methacryloyloxy)ethyl succinate (mMAOES), (*R*)-α-acryloyloxy-β,β-dimethyl-γbutyrolactone (AODMBA), triethylene glycol methyl ether methacrylate (TEGMA), isobornyl acrylate (iBoA), isodecyl methacrylate (iDMA), 2,2′-azobis (2-methylpropionitrile) (AIBN, 98%), 2,2-dimethoxy-2-phenylacetophenone (DMPA), 2,2′-azobis(4-methoxy-2,4-dimethylvaleronitrile (V70) and mercaptopropionic acid (MPA, 99%) were purchased from Sigma-Aldrich (monomer structures are given in Fig. S1†). Dodecanthiol (DDM, 98%) was acquired from Fluka. Cyclohexanone (99%) and tetrahydrofuran (THF, 99%) were used as solvents for the polymer synthesis while hexane, methanol and chloroform were the anti-solvents used for polymer precipitations, all supplied by Fisher Scientific. Potato dextrose agar (PDA) was obtained from Oxoid and potato dextrose broth (PDB) was supplied by Sigma-Aldrich. All the materials were used as received unless stated otherwise.

### Photo-polymerisation procedure

Photo induced Free Radical Polymerisation (FRP) *via* photoinitiation was performed in 96 well-plates. Monomer solutions were prepared at the concentration of 1 wt% with respect to monomer, using DMPA as the photoinitiator. Aliquots (100 μl) of these solutions were added into each well and the well plate was irradiated with UV (Blak-Ray XX-15L UV Bench Lamp; 230 V, ∼50 Hz, 15 W, 365 nm) for 2 hours, with O_2_ < 2000 ppm. The samples were dried at <50 mTorr for 7 days prior to further use/analysis.

### Thermal polymerisation procedure adapted to the 10 g scale

Thermally induced, thiol-mediated FRP at a 10 g scale was conducted as follows. Appropriate quantities of the relevant monomer and 2,2-azobis(2-methylpropionitrile) (AIBN) initiator (0.5 wt% with respect to the monomer) were introduced into the required volume of cyclohexanone with stirring. The thiol chain transfer agent, dodecyl mercaptan (DDM), was then added at a concentration of 1 mol% with respect to the monomer. The reaction vessel was submerged in an ice-bath and the contents were degassed by purging with argon for at least 1 h. To commence the reaction, the temperature was raised to 75 °C after placing the flask in an oil bath and once this temperature was achieved, the reaction was allowed to proceed for 18 h with continual stirring. Polymer was purified by precipitation of the crude reaction mixture into an excess of hexane/chloroform, where the typical non-solvent to reaction-medium ratio was 5 : 1 v/v. Finally, precipitated polymers were dried *in-vacuo* at 25 °C for at least 24 h. ^1^H-NMR spectroscopic analysis (see below) was performed on both the crude polymerisation solution to determine polymer conversion and the precipitates to establish the final homopolymer composition.

### Thermal polymerisation procedure adapted to the 250 g scale

The protocol for the synthesis of the homopolymers was similar to that at the 10 g scale. The appropriate quantities of the monomer(s) and V70 initiator (0.5 wt% with respect to the monomer) were introduced into the required volume of THF with stirring in a 3-neck round bottom flask. The thiol chain transfer agent, 3-mercaptopropionic acid (MPA), was added at a concentration of 1 mol% with respect to the monomer. As in the 10 g scale procedure the reaction vessel was degassed and raised to 40 °C, then in this case allowed to proceed for 5 h with continual stirring. To monitor the bulk temperature during the polymerisation, a thermometer was introduced into the reaction mixture through a septum and retained there for the entire reaction period. Polymer was purified by precipitation into an excess of hexane (pDEGEEA) or chloroform (pmMAOES) following the steps for the 10 g scale procedure.

### Polymer characterisation

#### Nuclear magnetic resonance (^1^H-NMR)

Nuclear Magnetic Resonance (NMR) spectra were recorded at 25 °C with Bruker AV400 and AV3400 spectrometers (400 MHz) using deuterated chloroform. Chemical shifts were assigned in parts per million (ppm). Samples were dissolved in CDCl_3_ and d-DMSO (mMAOES) to which chemical shifts are referenced (residual chloroform at 7.26 ppm and DMSO at 2.50 ppm). MestReNova 14.2.1 copyright 2021 (Mestrelab Research S. L.) was used for analysing the spectra.

#### Gel permeation chromatography (GPC)

GPC analysis, was performed using an Agilent 1260 Infinity instrument equipped with a double detector with the light scattering configuration. Two mixed C columns at 35 °C were employed, using THF as the mobile phase with a flow rate of 1 mL min^−1^. GPC samples were prepared in HPLC grade THF and filtered previous injection. Analysis was carried out using Astra software. The number and weight average molecular weights (*M*_n_ and *M*_w_) and polydispersity (*Đ*) were calculated using narrow standards of polymethyl methacrylate (PMMA) for the calibration curve.

#### Raman spectroscopy

Raman spectroscopy was performed using a Horiba Jobin Yvon LabRAM HR Raman microscope. Spectra were acquired using a 785 nm laser, a 10× objective and a 200 μm confocal pinhole. Spectra were detected using a Synapse CCD detector (1024 pixels) thermoelectrically cooled to −60 °C. Before spectra collection, the instrument was calibrated using the zero-order line and a standard Si(100) reference band at 520.7 cm^−1^. For single point measurements, spectra were acquired over a minimum range 475–1850 cm^−1^ with an acquisition time of 120 s and two accumulations to automatically remove the spikes due to cosmic rays and improve the signal to noise ratio. Spectra were collected from at least three random locations and averaged to give a mean spectrum.

#### Differential scanning calorimetry (DSC)

Polymer thermal properties were investigated by DSC (Q2000, TA Instruments, Leatherhead, UK), at a heating rate of 10 °C min^−1^. Data analysis was done with TRIOS software (version 4.4.0.40883). Pans with hermetical lids (TA Instruments, Brussels, Belgium) were used for sample analysis, with empty pans as the reference. Glass transitions were determined by performing two heating/cooling cycles between −90° and 200 °C.

#### Water contact angle (WCA)

Images of the sessile droplet profile of a droplet deposited on the specific test surface were recorded. From each image, the WCA was determined using the angle of intersection between a baseline representing the substrate and a Young-Laplace function fit to the drop profile. The Young-Laplace function models the droplet shape using a two radii of curvature. Measurements were taken over 3 areas for each polymer sample from which average and standard deviation values were calculated. A CAM200 instrument (KSV Instruments, Ltd) was used to dispense water droplets (2–12 μl depending on the experiment) onto each polymer sample. Ultrapure water was used for all WCA measurements (18.2 MΩ resistivity at 25 °C).

### Fungal strains and culture

Filamentous fungi used in this study were *Botrytis cinerea* SAR109940, *Zymoseptoria tritici* K4418, *Chaetomium globosum* ATCC6205, *Colletotrichum gloeosporioides* ATCC38237, *Aspergillus fumigatus* Af293, *Aureobasidium pullulans* ATCC15233 and *Trichoderma virens* ATCC9645. The fungi were routinely maintained and grown on potato dextrose agar (PDA) or potato dextrose broth (PDB) statically at room temperature.

### Assessment of fungal attachment and fungal growth

Similar to previous work,^20^ asexual fungal spores (100 μl of 2.5 × 10^6^ spores ml^−1^ in PDB), freshly isolated from 7-day old PDA plates, were transferred to polymer-coated or -uncoated (see above) 96-well polystyrene microtiter plates (Greiner Bio-One, Stonehouse, UK) and incubated statically for 6 hours at room temperature. Non-adherent spores were removed by three gentle washes with PBS, then 100 μl of fresh PDB was added to each well, and plates were incubated at room temperature up to a further 18 hours. The wells were washed three times with PBS, and the XTT reaction to quantify (metabolic activity of) attached fungi was initiated by adding XTT and menadione to final concentrations of 400 μg ml^−1^ and 25 μM in PBS, final volume 200 μl per well. After 6 hours, 100 μl of the reaction solution was transferred to fresh 96-well plates, and the absorbance was measured at 490 nm using a BioTek EL800 microplate spectrophotometer. To test for any effect of the materials on fungal growth, the fungi were cultivated in PDB in the polymer-coated wells, as above, but here for 14 days and with all washing steps omitted. Growth effects were assessed by image analysis or by observation where appropriate (the XTT reaction cannot be performed in PDB medium, and the fungi do not grow in PBS).

### Fungal infection of isolated wheat leaf segments

Polymer solutions [20% (w/v), prepared using 20% (v/v) isopropanol as solvent] were sprayed onto leaf segments, length 1.5 cm and width 1 cm, prepared from <15 day old Paragon lettuce cultivars. Leaf segments were placed onto 2% (w/v) water agar [sterile distilled water, 2% (w/v) agar (Sigma-Aldrich)] in square Petri plates (Greiner). Spores of *B. cinerea* were harvested from 7-day old PDA plates, washed twice with PDB, and adjusted to a concentration of 5 × 10^5^ spores ml^−1^ PDB. Once dried, leaf segments were infected with *B. cinerea* by spraying the spore suspension to the segments. Images were captured every day up to 3 days after infection to assess lesions visually and monitor progress of infection. To assess potential toxicity of polymers to the plant material, leaf segments were sprayed with the polymers but not infected with *B. cinerea*, followed by observation over time as above.

### Spore hydrophobicity

The method was adapted from Liauw *et al.*^24^ Spores were harvested from 7 day old PDA plates, washed twice with PBS and adjusted to a concentration of 2 × 10^7^ per ml in 500 μl PBS in glass vials. Toluene (500 μl; Fisher Scientific Ltd) was then added to the spore suspension and vortexed for 90 seconds prior to static incubation for 30 minutes at 37 °C to allow for phase separation of the largely immiscible toluene-water mixture. A 100 μl aliquot of the aqueous phase was removed and the spore concentration in this sample determined by counting with a haemocytometer. This final spore concentration (after toluene extraction) was subtracted from the initial spore concentration and the result divided by the initial spore concentration and multiplied by 100 to give the percentage of total spores that partitioned with the hydrophobic phase, thus giving the spore hydrophobicity (%) as described.^24^

### Field trial with wheat infected by *Z. tritici*

A fully randomised field trial was performed at the ADAS Rosemaund site, Herefordshire, UK, with a crop of the winter wheat variant Barrel, which is susceptible to *Z. tritici* infection. Trial plots were 6 m × 2 m in size with four replicate plots for each of five treatments and a control. Polymers mMAOES and DEGEEA, synthesised as described in “Thermal polymerisation procedure adapted to the 250 g scale”, were supplied at 5% and 10% (w/v), respectively, in a dH_2_O/isopropanol solution (80 : 20). Polymers were applied (as described below for all treatments) at three different time points corresponding with sequential emergence of the top three leaf layers during growth of the wheat (T1, 28/04/2022; T1.5, 09/05/2022; T2, 19/05/2022) (Table 1). (These three timings corresponded to the GS32, GS37 and GS39 wheat growth stages respectively).^25^ Fungicide programme 1 (Arizona fungicide only) was applied at the same time points of wheat growth as the polymers (T1, T1.5, T2) (Table 1). Fungicide programme 3 was designed to reflect a commercial fungicide program for this variety, whereas programme 2 was a lower input approach containing the triazole components of programme 3. In both programmes 2 and 3, different actives were applied at the key fungicide timings T1 and T2 (Table 1). Fungicide products were appropriately diluted with water (Table 1) and all treatments were applied using handheld plot sprayers with flat fan nozzles at 2 bar pressure and a rate of 200 l ha^−1^. At 8 and 10 weeks after the first spray application, percentage leaf-area diseased by *Z tritici* and green leaf area were estimated by independent assessors for each leaf layer, at four locations within each of the four plots per treatment. Wheat plant height was also measured by assessors on five primary tillers (*i.e.* five main shoots) per plot, at 13 weeks after the first spray application.

**Table 1:** Field Trial Treatment Schedule

**Treatment**	**Timings (T1, T1.5, T2; dates) and composition of repeated treatment applications to the crop during the trial** a			**Active ingredients (fungicides)**
	**T1 application;** 28 Apr 2022	**T1.5 application;** 09 May 2022	**T2 application;** 19 May 2022	
Control	Untreated	Untreated	Untreated	—
Polymer mMAOES^b^	mMAOES^c^	mMAOES	mMAOES	—
Polymer DEGEEA	DEGEEA	DEGEEA	DEGEEA	—
Fungicide programme 1	Arizona^d^ (1.0 L ha^−1^)	Arizona (1.0 L ha^−1^)	Arizona (1.0 L ha^−1^)	Folpet (a phthalimide compound)
Fungicide programme 2	Arizona (1.0 L ha^−1^) + Proline275 (0.36 L ha^−1^)	Untreated	Arizona (1.0 L ha^−1^) + Myresa (1.25 L ha^−1^)	Folpet, prothioconazole, mefentrifluconazole
Fungicide programme 3	Arizona (1.0 L ha^−1^) + Univoq (1.0 L ha^−1^)	Untreated	Arizona (1.0 L ha^−1^) + Revystar XE (1.25 L ha^−1^)	Folpet, prothioconazole, fenpicoxamid, mefentrifluconazole, fluxapyroxad

a Disease was assessed at 8 and 10 weeks after the date of the first (T1) treatment application.

b Full monomer names and structures are given in Fig. S1.†

c 5% and 10% (w/v) preparations of mMAOES and DEGEEA, respectively, were applied at rate 200 l ha^−1^.

d Fungicide proprietary names are given; active ingredients within each fungicide programme are listed in the final column. The rates refer to final equivalent rate of application of the undiluted fungicide products, after accounting for dilution and rate used for the spray application (200 l ha^−1^).

### Statistical analysis

Statistical analyses were carried out by student's *t* test, linear regression, Pearson correlation, or two-way analysis of variance (ANOVA), using Graphpad Prism (9.4.1) software and a minimum of three independent replicate values. Correlation analysis between normalised attachment for different fungi and water contact angle of polymer was performed using Graphpad Prism with an alpha value of 0.05.

## Results and discussion

### Synthesis of anti-attachment homo-polymers of interest

**Fig. 1:**
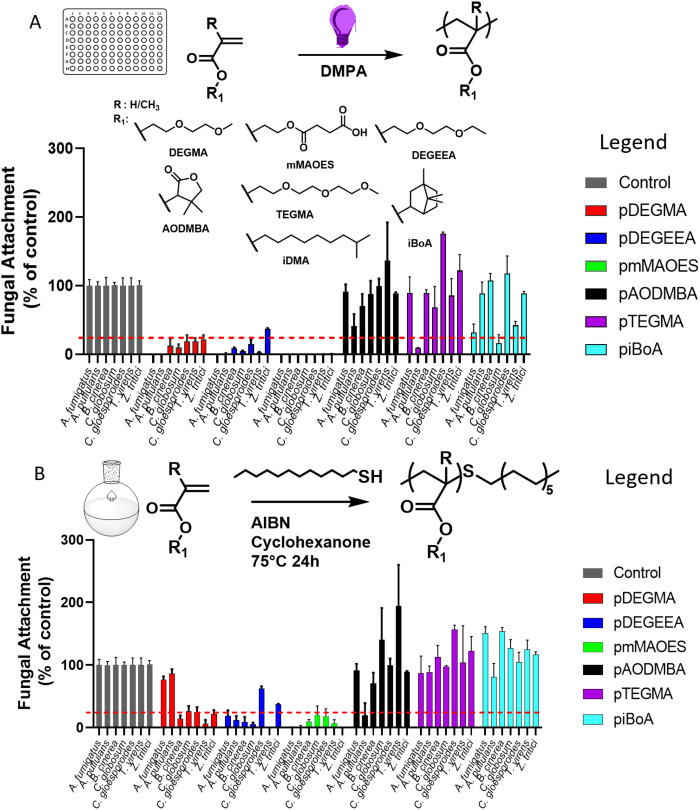
Certain homopolymers of interest reduce attachment of a diverse panel of filamentous fungi. Fungal attachment (according to XTT-reduction activity following removal of non-adhering spores) to photo-polymerised (A) or thermal polymerised (B) homopolymers, relative to uncoated polystyrene control (grey). An arbitrary threshold of 25% attachment relative to control is signified by the red dotted line. Fungi were tested in technical (*n* = 3) and biological (*n* = 3) replicates, with error bars denoting standard error of the mean (SETM) between biological replicate values.

Photo-cured homopolymers were produced by exposing mixtures consisting of monomer and photoinitiator to UV light, in polystyrene 96-well plates (Fig. 1A). The homopolymers utilised were selected for their anti-attachment properties defined by the previous results of Vallieres *et al.*^20^ and the photo-polymerisation strategy followed the procedure detailed in that report. To evaluate the level of monomer conversion to polymer achieved during the photo-curing process, micro-Raman spectroscopy was performed to calculate the degree of consumption (DC) of (meth)acrylate groups (eqn (S1)†).^26^ All wells showed a quantitative conversion, *i.e.*, a >99% DC of (meth)acrylate groups, with the exception of piBoA (DC = 91%) and pAODMBA (DC = 95%) (Fig. S2†). The lower DC's for these two homopolymers might be related to their high glass transition temperature (*T*_g_) (see Table 2) and the high viscosity (due to the high conversion) reducing the diffusion among the growing polymer chains during photo-curing.

**Table 2:** *M*_n_, *Đ*, and the physical characterisation of the thermally synthesised polymers (*T*_g_ and WCA). Conversions of all the obtained polymers were >90%. Reactions were performed using 1% mol of DDM as chain transfer agent and at a scale of 10 g of initial monomers

Polymer^a^	*M*_n_ b (g mol^−1^)	*Đ* b	*T*_g_ (°C)	WCA (°)
pDEGMA^c^	15 020	1.78	−38	40.6 ± 1.8
pTEGMA	17 290	2.10	−51	19.5 ± 1.8
pDEGEEA	7210	3.23	−55	28.6 ± 1.8
pAODMBA^d^	5100^c^	—	85	73.0 ± 1.7
pmMAOES	5000	1.30	11	49.0 ± 1.5
piDMA	25 380	1.68	−41	93.0 ± 1.5
piBoA	9833	1.42	92	99.0 ± 3.0

a Corresponding data for polymers prepared by photo-initiated free radical-polymerization are available in Vallieres *et al.*^20^

b *M*_n_ and *Đ* were calculated by GPC.

c Full monomer names and structures are given in Fig. S1.†

d *M*_n_ of pAODMBA was calculated *via*^1^H-NMR due to the poor solubility of this polymer in THF.

As high conversions were attained using a photo-polymerisation strategy adopted in prior study by the authors, this mode of polymerisation was used for initial screening of the monomers of interest. However, a photo-polymerisation strategy may not be well suited to applications, such as in agriculture, due to the necessity for spraying volatile monomers and the need to deliver sufficient UV exposure to initiate polymerisation.^27^ Consequently, the monomers of interest were also thermal polymerised in solvent solution using thiol-mediated polymerisation for comparison. Many commercial polymerisation processes achieve reaction control (*i.e.* molecular weight, dispersity, *etc*.) *via* the use of chain transfer agents (CTA), such as thiols.^28,29^ This allowed the final *M*_n_ of the polymers to be tailored to values ranging from 5000 to 20 000 g mol^−1^ to obtain an appropriate viscosity for a spraying process (*i.e.* a degree of polymerisation (DP) of ∼ 100 to 200). The complete range of homo-polymers produced by thermal polymerisation and their physical properties (*e.g. T*_g_, water contact angle) are presented in Table 2.

The polymer characterisation data demonstrated that the use of the dodecyl mercaptan (DDM) as a CTA successfully resulted in the synthesis of homopolymers with molecular weights lower than 26 000 g mol^−1^ and dispersity index values (*Đ*) of ≤2.00 (Table 2). Only pDEGEEA showed a *Đ* value greater than 3.00, which was attributed to a fast propagation rate during the polymerisation reducing the level of control by the thiol transfer agent. Thus, for future studies with this monomer, better kinetic control might require a higher CTA concentration or higher solvent/monomer ratio.^30,31^

The low glass transition temperatures (ranging between −35 °C and 11 °C, see Table 2) of some of the homopolymers synthesised from these key bio-instructive monomers (*i.e.* pDEGMA, pDEGEEA pTEGMA, piDMA and pmMAOES) meant that they were ‘liquid-like’ at application temperature. This physical property makes them easily miscible in organic solvents, water and/or mixtures of both,^32^ making these homopolymers more suitable for a crop formulation as they do not require many time-consuming steps for the preparation of solutions. However, this property would have to be balanced in application with the potential for the coating to display creep whilst in service, potentially leaving areas of the surface uncovered so open to colonisation.

### Assessment of a role for surface hydrophobicity in anti-attachment properties of polymers

Some studies investigating fungal or bacterial attachment have reported that surface hydrophobicity can be an influential factor in promoting or preventing attachment,^11,24,33,34^ although broader extrapolation of these observations has been questioned when tested on diverse libraries of material chemistry.^35^ Wettability is also a key consideration for the leaf surface in the application of crop protection chemicals, where efficacy depends on the coverage of aqueous solution and suspensions on leaf surfaces.^36^ The untreated wheat leaf water contact angle (WCA) was approximately 150° (Fig. S4C†), defining the surface to be super hydrophobic.^37^ Meanwhile, several of the homopolymers created in this study were found to be comparatively hydrophilic (*i.e.* WCA < 90°), as determined by WCA measurements made on dip-coated glass coverslips (Table 2) and also by the marked decrease in WCA of polymer-coated leaves (Fig. S4C†). This was attributed to the molecular structures of the monomers, where pDEGEEA, pDEGMA and pTEGMA each contain two or three ethylene glycol units and pmMAOES and pAODMBA have polar side chains. In contrast, piDMA (with a long alkyl chain) and piBoA (with bulky ring pendant group) have contact angle values >90° (Table 2).

It was assumed that for a polymer-coated leaf surface, the arrangement of the homopolymers involves the hydrophobic polymer backbone interacting preferentially with long chain hydrocarbons and tri-terpenes, main components of the leaf wax, so leaving the hydrophilic pendant groups oriented toward the external environment; thus, the leaf surface would now present as a more hydrophilic surface (that may discourage the attachment of organisms seeking a hydrophobic surface).^31,38^ To explore whether hydrophobic/hydrophilic interactions between the fungal spores and the (meth)acrylate polymers had a possible influence on attachment, spore hydrophobicities for different fungi and polymer surface WCAs were compared with levels of attachment (Fig. S3†). No significant correlation was found between spore hydrophobicity and attachment either across a range of polymers (*p* > 0.05) or when examined on a polymer by polymer basis (Fig. S3B†). This suggested that hydrophobic interactions might not be a primary determinant of the level of fungal attachment. On the other hand, a significant relationship was found between polymer-WCAs and attachment across the test fungi (*p* < 0.05), with the majority of individual fungal species giving *R*^2^ > 0.5 (Fig. S3C†). All the fungi showed low attachment with the most hydrophilic materials (WCA ≤50°), suggesting that this level of hydrophilicity deters fungal attachment. The aim of this study was not to give a deep understanding of the mechanism of action of these materials; rather, their potential for application as crop protection chemicals. Nevertheless, these preliminary results suggest that WCA of the coatings could prove one useful tool for prediction of attachment properties.

### Assessment of polymer performance in resisting fungi

Both photo- and thermal-polymerised polymers were tested for their anti-attachment performance against a range of filamentous fungi associated with disease of crops or humans, or with colonisation of material surfaces. Photo-polymerised pDEGMA, pDEGEEA and pmMAOES were observed to reduce attachment of nearly all the test fungi to less than 25% of the attachment supported by the polystyrene control (Fig. 1A).

**Fig. 2:**
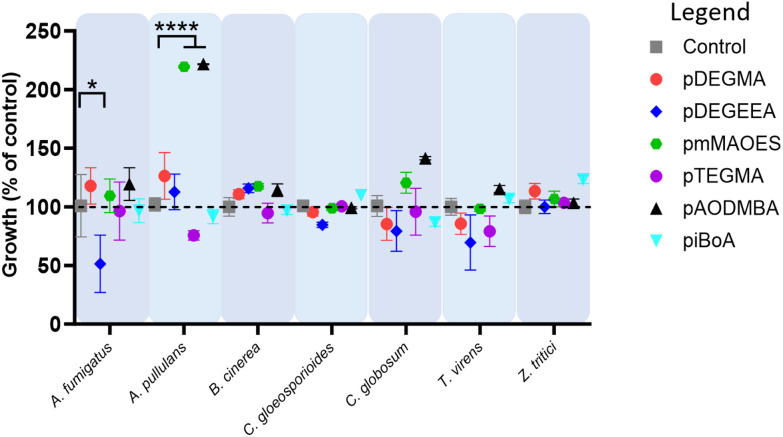
Absence of toxic effects of the homopolymers on fungal growth. Fungal growth after 14 days in PDB medium in wells coated with the different polymers, relative to control growth in uncoated wells. Fungi were tested in technical (*n* = 3) and biological (*n* = 3) replicates, with error bars denoting standard error of the mean (SETM) between biological replicate values. *p < 0.05 and ****p < 0.0001 according to the Kruskall–Wallace test.

Similar results were obtained for thermal polymerised pDEGMA, pDEGEEA and pmMAOES, which reduced relative attachment to less than 25% in most (pDEGMA, pDEGEEA) or all seven (pmMAOES) of the tested fungal species (Fig. 1B). Meanwhile, pAODMBA, pTEGMA and piBoA, were much less effective at reducing attachment, with only the relative attachment of *A. pullulans* decreased to below 25% by thermal polymerised pAODMBA for example (Fig. 1B). In fact, several of the fungi were found to attach more strongly to these polymers than to the polystyrene control. To corroborate that low attachment was not due to any toxic effect related directly to the polymers, fungi were cultured in the presence of the polymers for 14 days within a 96-well plate format in PDB medium and total growth of fungi within the medium itself was compared with that of fungi cultured in uncoated-polystyrene control wells. No statistically significant decrease in fungal growth yield *versus* the control was observed, except in the case of *A. fumigatus* growing in the presence of DEGEEA (Fig. 2; *p* < 0.05). Overall, these results corroborate previous studies, which indicated a general lack of toxicity of these polymers to fungi, bacteria and mammalian cells.^20,39–41^ In fact, it was noted that the total growth yield of *A. pullulans* within the medium was >2-fold greater in pmMAOES- or pAODMBA-coated wells when compared to the uncoated wells (*p* < 0.001), suggesting the possibility that *A. pullulans* might use these polymers as a nutrient source. *A. pullulans* is known to secrete an arsenal of digestive enzymes that are effective at breaking down materials for nutrient acquisition.^42^ Overall, these data highlighted the non-toxic nature and step-change anti-attachment efficacy of certain homopolymers from the initial set, namely pDEGMA, pDEGEEA, and pmMAOES, against a range of phylogenetically distinct and problematic fungi with diverse spore morphologies, including the major wheat pathogen *Z. tritici*. Thus, these were highlighted as the potential candidate polymers to be moved into scale-up synthesis development and for deployment in a field-trial.

### Polymerisation scale-up and field trial of efficacy on wheat against infection by *Z. tritici*

Following the above physical and biological characterisation of the homo-polymers library, the synthesis of two of the most promising polymers, pDEGEEA and pmMAOES, was scaled-up to 250 g batch using thermal polymerisation. Thermal polymerisation was used because of the similar anti-attachment efficacy as with photo-polymerisation (Fig. 1A and B), but photo-polymerisation may be less suited to applications as discussed above. pDEGEEA and pmMAOES were selected based on the *in vitro* anti-attachment properties against the wheat pathogen *Z. tritici*^20^ (Fig. 1) and in leaf-infection assays against *B. cinerea* (Fig. S4A†). The scaled FRP synthesis method employed the same CTA concentration (1% mol) used for the small-scale production of polymer in Table 2. In this set of reactions, the temperature was reduced to 40 °C with the aim of using THF as solvent as it has a lower boiling point than cyclohexanone used in the small scale reactions. The use of THF facilitated the purification steps as the reaction mixture was concentrated prior to precipitation. Considering the lower temperature applied for the reaction, V70 was employed as the thermal initiator and the polymerisation was left for 5 h considering that the half-time of this initiator at 40 °C is around 2.5 h. In this larger scale synthesis, mercaptopropionic acid (MPA) was used as the CTA for control of the thiol mediated FRP, because its molecular structure contains a carboxylic acid moiety (Fig. S5A† for polymerisation mechanism and chemical structure of the CTA). It was considered that this functional group would improve the retention on leaf wax surfaces, which are rich in hydroxyl groups due to the presence of alcohols and terpinol-based molecules.^36,43–45^ The data in Table S1† show that the 250 g scale polymers exhibited *M*_n_ values and conversion efficiencies very similar to those of the smaller-scale synthesis. This straight-forward transition from small- to large-scale is characteristic of the high performance of the thiol-mediated FRP method. This is important as it supports the viability of these polymers for potential crop protection application at field and agro-food chain levels.

The anti-attachment properties of these MPA controlled homopolymers were initially corroborated against two test fungi (*B.cinerea* and *C.gloeosporioides*). Both homopolymers showed a reduction of attachment for both fungal species, with attachment ≤25% relative to the polystyrene control (Fig. S5B†), supporting the use of MPA for the scale-up polymerisations.

**Fig. 3:**
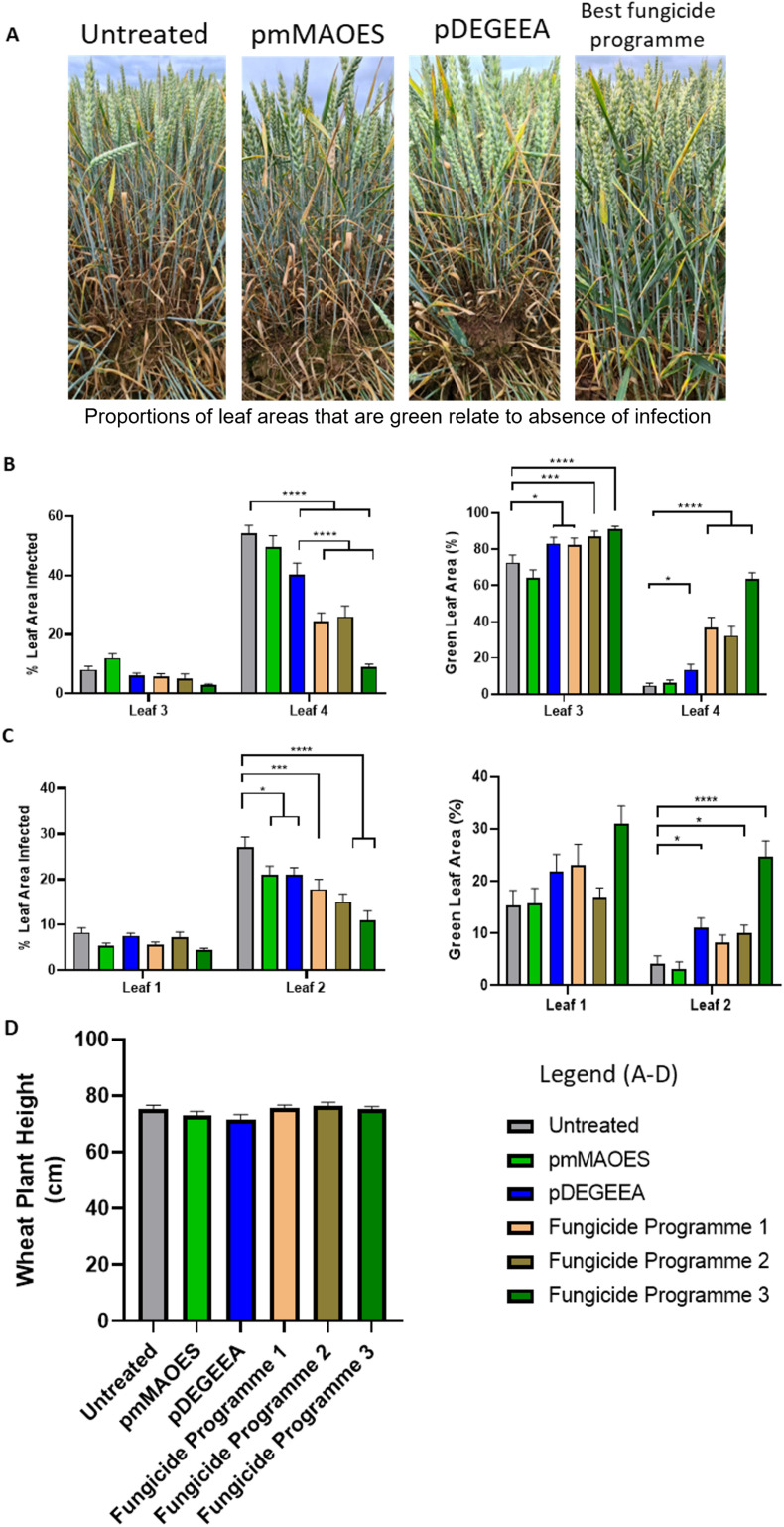
Wheat infection by *Z. tritici* in polymer- or fungicide-treated plants. (A) Representative images of wheat plants at 10 weeks after first treatment. From left to right: Untreated, and mMAOES-, DEGEEA-, or fungicide programme 3-treated. (B) Percentage leaf-area infected (left panel) and green leaf areas (GLA; right) in leaves 3 and 4, measured 8 weeks post-application of first treatments. (% infected and GLA values do not necessarily add up to 100% due to effects of other factors, such as leaf senescence with aging or insect damage). Wheat leaves were either untreated (control) or sprayed using treatments indicated in the mini-legend (as detailed in Table 1). (C) The same parameters as in (B) for leaves 1 and 2 measured after 10 weeks. (D) Wheat plant height measured 13 weeks after first treatments, as indicator of potential phytotoxicity. *p < 0.05; ***p < 0.001; ****p < 0.0001, according to the Kruskall–Wallace test. Mean values are shown ± SETM.

We considered the potential application of these polymers for crop protection from fungal phytopathogens in the field, so reducing dependence on fungicides. Although fungicide use is an effective fungal control method, it can have environmental impacts including destruction of native fungal species important for maintaining soil and ecological health and it also selects for emergence of fungicide resistant strains.^46,47^*Z. tritici* is a common plant pathogen that is typically tackled with fungicide applications.^8,46^ A field trial was performed to investigate natural infection by *Z. tritici* of wheat treated with pmMAOES or pDEGEEA. For comparison, three commercial fungicide programmes were tested in parallel (Table 1). The timings of three sequential spray-applications of pmMAOES and pDEGEEA, to treat leaves as they emerged at different wheat growth stages, matched those used for fungicide programme 1 (Arizona only). The other two programmes each included two spray applications (Table 1) which followed the standard treatment procedure for these particular fungicides. At 8 and 10 weeks after the first treatment application (*i.e.*, 8 and 10 weeks after T1; Table 1), both the percentage area of leaves infected by *Z. tritici* and the percentage green-leaf area (GLA) were estimated ‘blind’ through visual inspection by independent assessors (Fig. 3).

Inspection of the control set of plants at 8 weeks showed that the oldest leaves (referred to as leaf 4) had the most developed infections (Fig. 3B). The pDEGEEA treated plants showed a ∼25% reduction of *Z. tritici* infection (*p* < 0.001) (measured by percentage of leaf area infected) and a 2.9-fold increase in the GLA (*p* < 0.01) (Fig. 3B). Meanwhile, pmMAOES did not have a statistically significant on the level of *Z. tritici* infection established upon these leaves after 8 weeks. By comparison, the three commercial fungicide programmes were more effective at reducing *Z. tritici* infection than either polymer in the case of leaf 4 at this stage, but not in the case of leaf 3. The level of infection was lower in leaf 3 than leaf 4 at 8 weeks, but the GLA data at this stage showed that pDEGEEA performed at least as well as the Arizona fungicide (programme 1) and not significantly differently from the other commercial programmes (*p* > 0.05) (Fig. 3B).

By the tenth week after first treatment, leaves 3 and 4 were too infected/senesced to be usefully scored. Leaf 2 had reasonable levels of infected leaf area by week 10 and here both pDEGEEA and pmMAOES decreased the level of *Z. tritici*-infected area by >20% (*p* < 0.05) (Fig. 3C). An effect of pmMAOES was not discernible by the GLA measurement, but pDEGEEA increased the GLA by 2.6-fold in leaf 2 (*p* < 0.05). This effect of pDEGEEA was at least as large as the GLA increase delivered by either of the lower input commercial programmes (1 and 2), and better than the Arizona fungicide (programme 1) which did not give a significant benefit *versus* control (*p* > 0.05) (Fig. 3C).

According to assessors, some plots where DEGEEA was applied appeared slightly lighter in colour. However, quantitative assessment for phytotoxicity according to plant height (measured after 13 weeks) revealed that there were no statistically-significant differences in the heights of the pmMAOES- or pDEGEEA-treated plants compared with untreated control plants (Fig. 3D). This finding was consistent with the leaf based assays (Fig. S4B†) and the prior laboratory based reports of low toxicity generally to filamentous fungi (Fig. 2), bacteria and mammalian cells.^20,40,48^ It could be useful in future work to monitor additional wheat parameters, such as crop yields, as the present trials were not taken to yield.

## Conclusion

This field trial has provided the first real-world scale exemplification that the pDEGEEA and, to a lesser extent, pmMAOES (meth)acrylate homopolymers are effective at reducing the infection (up to 26%) by a filamentous fungus (*Z. tritici*) of wheat plants using a non-fungicidal mechanism. Furthermore, application of these homopolymers did not have a significant effect on the wheat plants’ overall growth. Importantly, from a commercialisation perspective, the demonstration that the polymers could be scaled up effectively should enable potential applications, such as in crop protection, to be viable. For application purposes, the materials are appropriately biodegradation-resistant and wash resistant.^20^ Material properties and precedents supporting safe use of these materials in the field are summarised in the Introduction. Nevertheless, if desirable in the future for environmental reasons, the polymers could be made more biodegradable by introducing degradable linking groups.

These results offer a promising step towards developing actives-free materials for control of problematic fungi that threaten food security. Fungi also cause important diseases in humans, food spoilage and materials degradation. Thus, the fungal anti-attachment materials have potential to deliver benefit in a wide range of applications. In this trial the pmMAOES and pDEGEEA applications did not include active fungal inhibitors nor had these formulations been optimised for industrial, spray-based applications to wheat. Therefore, while the results provide strong proof-of-concept for slowing or preventing *Z. tritici* infection of wheat crops, there is also considerable scope for further development of the polymer formulations with a view to improving efficacy through modification of the polymer concentration, spray pressure and droplet size, application timings, addition of acid groups to improve leaf retention, and further explorations into co-polymerisation formulations to optimise the polymer *T*_g_.^49–52^ Finally, this study achieved successful translation of a polymer identified from a high throughput screening approach using *in vitro* bioassay data and methods to a small scale field trial, hence, validating and supporting the screening experiment conclusions and the scale up methodologies.

## Conflicts of interest

There are no conflicts of interest.

## Supplementary Material

GC-025-D3GC01911J-s001

## References

[cit1] Hyde K. D. (2019). *et al.*, The Amazing Potential of Fungi: 50 ways we can exploit fungi industrially. Fungal Diversity.

[cit2] Meyer V. (2016). *et al.*, Current challenges of research on filamentous fungi in relation to human welfare and a sustainable bio-economy: A White Paper. Fungal Biol. Biotechnol..

[cit3] Whitehead K. A., Deisenroth T., Preuss A., Liauw C. M., Verran J. (2022). Lateral force removal of fungal spores to demonstrate how surface properties affect fungal spore retention. Philos. Trans. R. Soc., A.

[cit4] Davies C. R., Wohlgemuth F., Young T., Violet J., Dickinson M., Sanders J. W., Vallieres C., Avery S. V. (2021). Evolving challenges and strategies for fungal control in the food supply chain. Fungal Biol. Rev..

[cit5] Fisher M. C. (2020). *et al.*, Threats posed by the fungal kingdom to humans, wildlife, and agriculture. MBio.

[cit6] Daly P. (2021). *et al.*, From lignocellulose to plastics: knowledge transfer on the degradation approaches by fungi. Biotechnol. Adv..

[cit7] Anonymous (2017). Stop neglecting fungi. Nat. Microbiol..

[cit8] Fones H., Gurr S. (2015). The impact of *Septoria tritici* blotch disease on wheat: an EU perspective. Fungal Genet. Biol..

[cit9] Fisher M. C., Hawkins N. J., Sanglard D., Gurr S. J. (2018). Worldwide emergence of resistance to antifungal drugs challenges human health and food security. Science.

[cit10] Wessels J. G. H. (1996). Fungal hydrophobins: proteins that function at an interface. Trends Plant Sci..

[cit11] Vasselli J. G., Shaw B. D. (2022). Fungal spore attachment to substrata. Fungal Biol. Rev..

[cit12] Cavalheiro M., Teixeira M. C. (2018). *Candida*, biofilms: threats, challenges, and promising strategies. Front. Med..

[cit13] Cámara M., Green W., MacPhee C. E., Rakowska P. D., Raval R., Richardson M. C., Slater-Jefferies J., Steventon K., Webb J. S. (2022). Economic significance of biofilms: a multidisciplinary and cross-sectoral challenge. npj Biofilms Microbiomes.

[cit14] O'Driscoll A., Kildea S., Doohan F., Spink J., Mullins E. (2014). The wheat-*Septoria* conflict: a new front opening up?. Trends Plant Sci..

[cit15] Vermeulen E., Lagrou K., Verweij P. E. (2013). Azole resistance in *Aspergillus fumigatus*: a growing public health concern. Curr. Opin. Infect. Dis..

[cit16] Qiu H., Feng K., Gapeeva A., Meurisch K., Kaps S., Li X., Yu L., Mishra Y. K., Adelung R., Baum M. (2022). Functional polymer materials for modern marine biofouling control. Prog. Polym. Sci..

[cit17] Barde M., Davis M., Rangari S., Mendis H. C., De La Fuente L., Auad M. L. (2018). Development of antimicrobial-loaded polyurethane films for drug-eluting catheters. J. Appl. Polym. Sci..

[cit18] Magennis E. P., Hook A. L., Davies M. C., Alexander C., Williams P., Alexander M. R. (2016). Engineering serendipity: high-throughput discovery of materials that resist bacterial attachment. Acta Biomater..

[cit19] Hook A. L., Anderson D. G., Langer R., Williams P., Davies M. C., Alexander M. R. (2010). High throughput methods applied in biomaterial development and discovery. Biomaterials.

[cit20] Vallieres C., Hook A. L., He Y., Cuzzucoli Crucitti V., Figueredo G., Burroughs L., Winkler D. A., Wildman R. D., Irvine D. J., Alexander M. R., Avery S. V. (2020). Discovery of (meth)acrylate polymers that resist colonization by fungi associated with pathogenesis and biodeterioration. Sci. Adv..

[cit21] Oliver R. (2022). Globalizing plant health. Plant Pathol..

[cit22] Hook A. L., Chang C. Y., Yang J., Atkinson S., Langer R., Anderson D. G., Davies M. C., Williams P., Alexander M. R. (2013). Discovery of novel materials with broad resistance to bacterial attachment using combinatorial polymer microarrays. Adv. Mater..

[cit23] Cuzzucoli Crucitti V., Ilchev A., Moore J. C., Fowler H. R., Dubern J. F., Sanni O., Xue X., Husband B. K., Dundas A. A., Smith S., Wildman J. L., Taresco V., Williams P., Alexander M. R., Howdle S. M., Wildman R. D., Stockman R. A., Irvine D. J. (2023). Predictive molecular design and structure–property validation of novel terpene-based: sustainably sourced bacterial biofilm-resistant materials. Biomacromolecules.

[cit24] Liauw C. M., Slate A. J., Butler J. A., Wilson-Nieuwenhuis J. S. T., Deisenroth T., Preuss A., Verran J., Whitehead K. A. (2020). The effect of surface hydrophobicity on the attachment of fungal conidia to substrates of polyvinyl acetate and polyvinyl alcohol. J. Polym. Environ..

[cit25] Wheat growth guide, https://projectblue.blob.core.windows.net/media/Default/Imported

[cit26] Henning I., Woodward A. W., Rance G. A., Paul B. T., Wildman R. D., Irvine D. J., Moore J. C. (2020). A click chemistry strategy for the synthesis of efficient photoinitiators for two-photon polymerization. Adv. Funct. Mater..

[cit27] Yamada B. and Zetterlund P. B., General chemistry of radical polymerization, in Handbook of Radical Polymerization, ed. K. Matyjaszewski and T. P. Davis, John Wiley & Sons, Hoboken, 2003, pp. 117–186

[cit28] Matyjaszewski K. and Davis T. P., Handbook of Radical Polymerization, John Wiley & Sons, Hoboken, 2002

[cit29] Wang J.-S., Matyjaszewski K. (1995). Controlled/"living" radical polymerization. Atom transfer radical polymerization in the presence of transition-metal complexes. J. Am. Chem. Soc..

[cit30] Heuts J. P. A., Muratore L. M., Davis T. P. (2000). Preparation and characterization of oligomeric terpolymers of styrene, methyl methacrylate and 2-hydroxyethyl methacrylate: a comparison of conventional and catalytic chain transfer. Macromol. Chem. Phys..

[cit31] Cuzzucoli Crucitti V., Contreas L., Taresco V., Howard S. C., Dundas A. A., Limo M. J., Nisisako T., Williams P. M., Williams P., Alexander M. R., Wildman R. D., Muir B. W., Irvine D. J. (2021). Generation and characterization of a library of novel biologically active functional surfactants (surfmers) using combined high-throughput methods. ACS Appl. Mater. Interfaces.

[cit32] Dudowicz J., Freed K. F., Douglas J. F. (2005). The glass transition temperature of polymer melts. J. Phys. Chem. B.

[cit33] Hamer J. E., Howard R. J., Chumley F. G., Valent B. (1988). A mechanism for surface attachment in spores of a plant pathogenic fungus. Science.

[cit34] Whitehead K. A., Liauw C. M., Wilson-Nieuwenhuis J. S. T., Slate A. J., Deisenroth T., Preuss A., Verran J., Whitehead K. A., Liauw C. M., Wilson-Nieuwenhuis J. S. T., Slate A. J., Deisenroth T., Preuss A., Verran J. (2020). The effect of the surface properties of poly(methyl methacrylate) on the attachment, adhesion and retention of fungal conidia. AIMS Bioeng..

[cit35] Alexander M. R., Williams P. (2017). Water contact angle is not a good predictor of biological responses to materials. Biointerphases.

[cit36] Taylor P. (2011). The wetting of leaf surfaces. Curr. Opin. Colloid Interface Sci..

[cit37] Feng L., Li S., Li Y., Li H., Zhang L., Zhai J., Song Y., Liu B., Jiang L., Zhu D. (2002). Super-hydrophobic surfaces: from natural to artificial. Adv. Mater..

[cit38] Dundas A. A., Cuzzucoli Crucitti V., Haas S., Dubern J. F., Latif A., Romero M., Sanni O., Ghaemmaghami A. M., Williams P., Alexander M. R., Wildman R., Irvine D. J. (2020). Achieving microparticles with cell-instructive surface chemistry by using tunable co-polymer surfactants. Adv. Funct. Mater..

[cit39] Mikulskis P., Hook A., Dundas A. A., Irvine D., Sanni O., Anderson D., Langer R., Alexander M. R., Williams P., Winkler D. A. (2018). Prediction of broad-spectrum pathogen attachment to coating materials for biomedical devices. ACS Appl. Mater. Interfaces.

[cit40] Singh T., Hook A. L., Luckett J., Maitz M. F., Sperling C., Werner C., Davies M. C., Irvine D. J., Williams P., Alexander M. R. (2020). Discovery of hemocompatible bacterial biofilm-resistant copolymers. Biomaterials.

[cit41] He Y., Abdi M., Trindade G. F., Begines B., Dubern J. F., Prina E., Hook A. L., Choong G. Y. H., Ledesma J., Tuck C. J., Rose F. R. A. J., Hague R. J. M., Roberts C. J., De Focatiis D. S. A., Ashcroft I. A., Williams P., Irvine D. J., Alexander M. R., Wildman R. D. (2021). Exploiting generative design for 3D printing of bacterial biofilm resistant composite devices. Adv. Sci..

[cit42] Chi Z., Ma C., Wang P., Li H. F. (2007). Optimization of medium and cultivation conditions for alkaline protease production by the marine yeast *Aureobasidium pullulans*. Bioresour. Technol..

[cit43] van Maarseveen C., Jetter R. (2009). Composition of the epicuticular and intracuticular wax layers on *Kalanchoe daigremontiana* (Hamet et Perr. de la Bathie) leaves. Phytochemistry.

[cit44] Lin J., Zhu M., Wu X., Zheng C., Liu Z., Wang Q., Lu D., He Q., Chen X. (2016). Microwave-assisted synthesis of trisiloxane superspreader and its superspreading behavior on plant leaves surfaces. Colloids Surf., A.

[cit45] Peirce C. A. E., Priest C., McBeath T. M., McLaughlin M. J. (2016). Uptake of phosphorus from surfactant solutions by wheat leaves: spreading kinetics, wetted area, and drying time. Soft Matter.

[cit46] Van Den Berg F., Van Den Bosch F., Paveley N. D. (2013). Optimal fungicide application timings for disease control are also an effective anti-resistance strategy: a case study for *Zymoseptoria tritici (Mycosphaerella graminicola)* on wheat. Phytopathology.

[cit47] Cheng G., Ning J., Ahmed S., Huang J., Ullah R., An B., Hao H., Dai M., Huang L., Wang X., Yuan Z. (2019). Selection and dissemination of antimicrobial resistance in agri-food production. Antimicrob. Resist. Infect. Control.

[cit48] Hook A. L., Chang C. Y., Yang J., Luckett J., Cockayne A., Atkinson S., Mei Y., Bayston R., Irvine D. J., Langer R., Anderson D. G., Williams P., Davies M. C., Alexander M. R. (2012). Combinatorial discovery of polymers resistant to bacterial attachment. Nat. Biotechnol..

[cit49] Peirce C. A. E., McBeath T. M., Priest C., McLaughlin M. J. (2019). The timing of application and inclusion of a surfactant are important for absorption and translocation of foliar phosphoric acid by wheat leaves. Front. Plant Sci..

[cit50] Grillo R., Mattos B. D., Antunes D. R., Forini M. M. L., Monikh F. A., Rojas O. J. (2021). Foliage adhesion and interactions with particulate delivery systems for plant nanobionics and intelligent agriculture. Nano Today.

[cit51] Makepeace D. K., Locatelli P., Lindsay C., Adams J. M., Keddie J. L. (2018). Colloidal polymer composites: are nano-fillers always better for improving mechanical properties?. J. Colloid Interface Sci..

[cit52] Carvalho F. K., Antuniassi U. R., Chechetto R. G., Mota A. A. B., de Jesus M. G., de Carvalho L. R. (2017). Viscosity, surface tension and droplet size of sprays of different formulations of insecticides and fungicides. Crop Prot..

