# Supplementary material for: A potential alternative to fungicides using actives-free (meth)acrylate polymers for protection of wheat crops from fungal attachment and infection
Source: Green Chem. 2023 Aug 17;25(21):8558–69. doi: 10.1039/d3gc01911j (PMC10614722; doi:10.1039/d3gc01911j)
Supplement: GC-025-D3GC01911J-s001 [file GC-025-D3GC01911J-s001.pdf]

Electronic Supplementary Material (ESI) for Green Chemistry.
This journal is © The Royal Society of Chemistry 2023

Supplementary Information:

A Potential Alternative to Fungicides using Actives-Free (Meth)Acrylate Polymers for Protection of Wheat Crops from Fungal Attachment and Infection

Liam A. Crawford,[a±] Valentina Cuzzucoli Crucitti,[b±] Amy Stimpson,[b,] Chloe Morgan,[d] Jonathan Blake,[d] Ricky D. Wildman,[b] Andrew L. Hook,[b] Morgan R. Alexander,[c] Derek J. Irvine,[b,*] Simon V. Avery[a,*]

[a]*School of Life Sciences, University Park, University of Nottingham, Nottingham NG7 2RD*
[b]*Centre for Additive Manufacturing, Department of Chemical and Environmental Engineering, University Park, University of Nottingham, Nottingham, NG7 2RD*
[c]*Advanced Materials and Healthcare Technologies, School of Pharmacy, University Park, University of Nottingham, Nottingham, NG7 2RD*
[d]*RSK ADAS Ltd, Rosemaund, Preston Wynne, Hereford, HR1 3PG*

$$DC = \left[1 - \frac{(A_{c=c}/A_{C=O})polymer}{(A_{c=c}/A_{C=O})monomer} \right] \times 100$$

Equation S1. Degree of Consumption (DC) of (meth)acrylate groups was determined by measuring the change in the ratio of peak areas associated with the C=C (≈1634 cm^{-1}) and C=O (≈1720 cm^{-1}) bonds before and after polymerization, according to Equation S1. In the equation, $(A_{c=c}/A_{C=O})_{monomer}$ and $(A_{c=c}/A_{C=O})_{polymer}$ refer to the integrated intensity of Raman peaks assigned to C=C and C=O groups of the (meth)acrylate functionality of the polymer and the related monomer. As a consequence of the polymerisation/crosslinking, it is assumed that the intensity of the C=C in the polymerised specimens is reduced as C-C bonds are formed with the formation of the polymer backbone. As C=O groups are not affected during the curing process, the intensity of this peak remains constant in both the monomer and polymer spectra, therefore serving as an internal reference.

Table S1. M_n, Đ, and % Conversion for scaled-up synthesis of homopolymers. Reactions were performed using 1% mol of MPA and at a scale of 250 g of initial monomer.

Entry	Conversion[a]	M_n (g mol^{-1})[b]	Đ[b]
pmMAOES	91%	8 100	1.84
pDEGEEA	98%	7 200	2.29

[a)]conversions were calculated by ^{1}H-NMR.[b)]M_n and Đ were calculated by GPC.

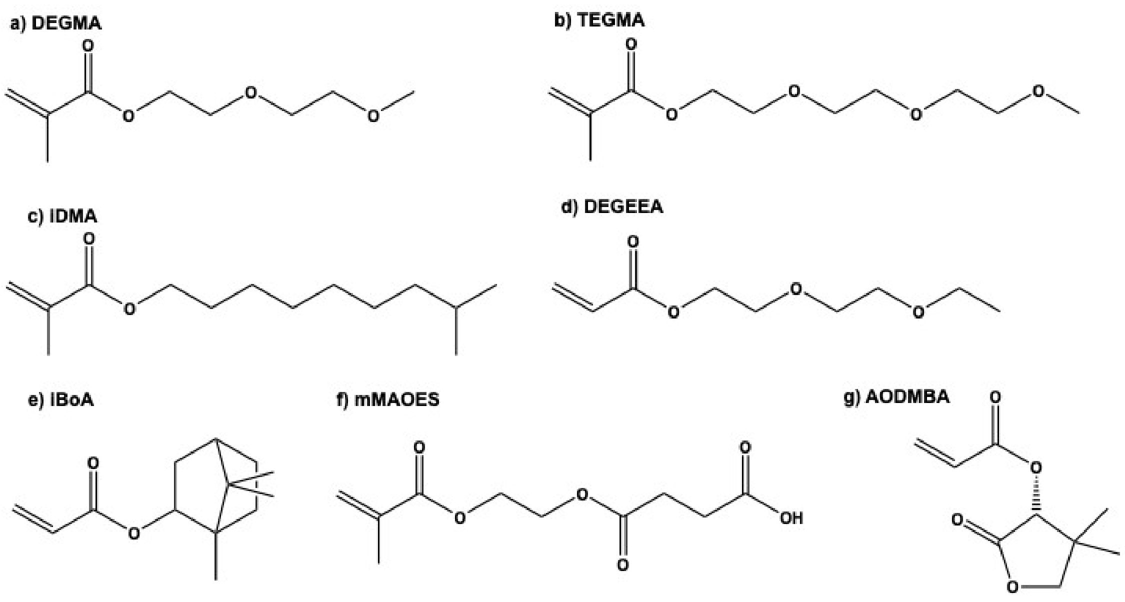

Figure S1. Structures of monomers used in this study. a) di(ethylene glycol) methyl ether methacrylate, b) triethylene glycol methyl ether methacrylate, c) isodecyl methacrylate, d) diethylene glycol ethyl ether acrylate, e) isobornyl acrylate, f) mono-2-(methacryloyloxy)ethyl succinate, and g) (R)-α-acryloyloxy-β,β-dimethyl-γbutyrolactone

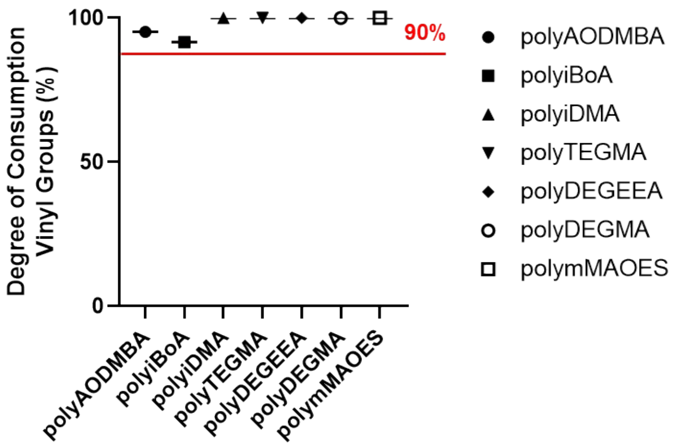

Figure S2. Degree of Consumption (DC) of vinyl groups values for each photo-polymerised homopolymer. DC values (n=3) are derived from the micro-Raman Spectroscopy analysis of the photo-cured homopolymers in 96 well-plate formats.

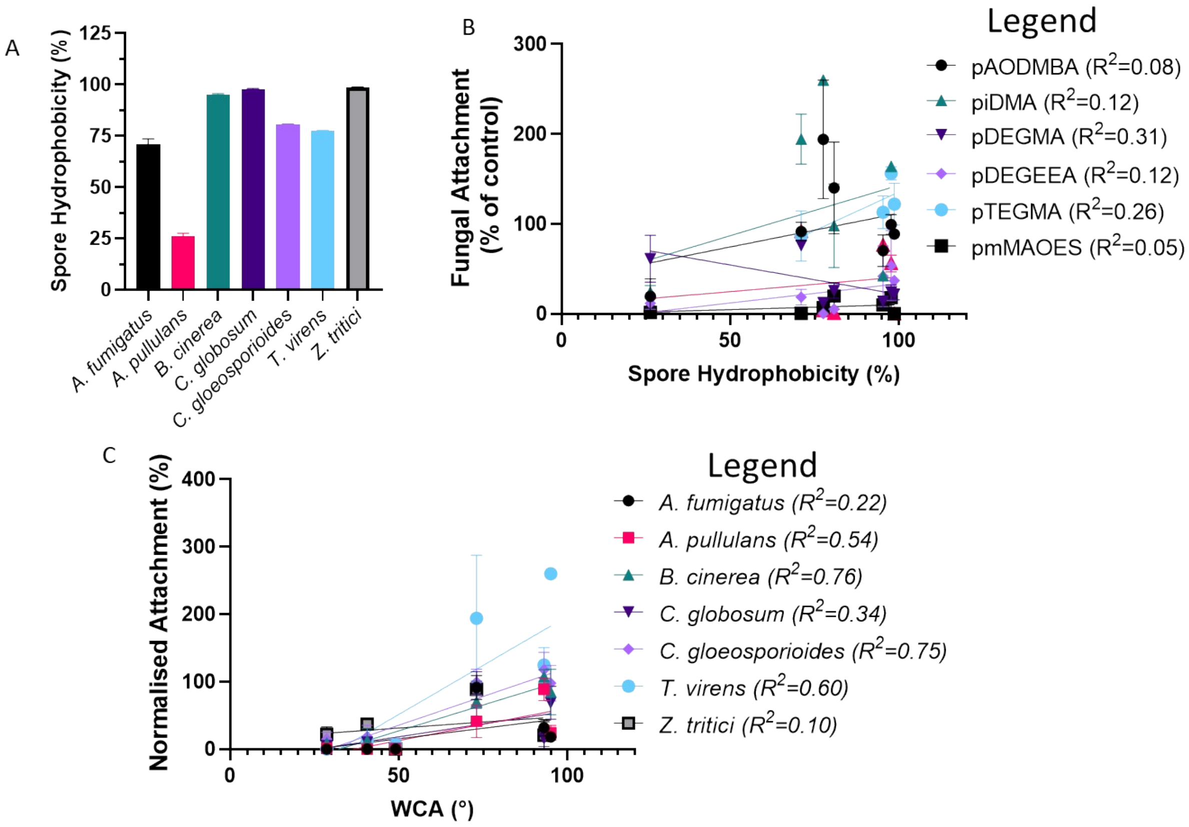

Figure S3. Fungal attachment versus spore and polymer hydrophobicities**.** (A) Spore surface hydrophobicity measured by phase-separation, with the proportion of spores in the hydrophobic phase reflecting hydrophobicity (%), from three biological replicates ± SETM. (B) Mean attachment (% of polystyrene control) of each fungus for each polymer plotted versus spore hydrophobicity. Points are means from three biological replicates ± SETM. Linear regressions shown for each polymer individually all gave $R^2 \leq 0.31$. There was no significant overall correlation between spore hydrophobicity and fungal attachment (p=0.15 R^2=0.36; Pearson's rank correlation coefficient; not shown). (C) Attachment (%) for each fungal species plotted against water contact angle (WCA) measured on all polymers. Linear regressions (shown) gave R^2 >0.5 for most of the fungal species individually. There was also a significant relationship between surface WCA and attachment across all the fungi (p=0.03 R^2=0.71); Pearson's rank correlation coefficient, not shown).

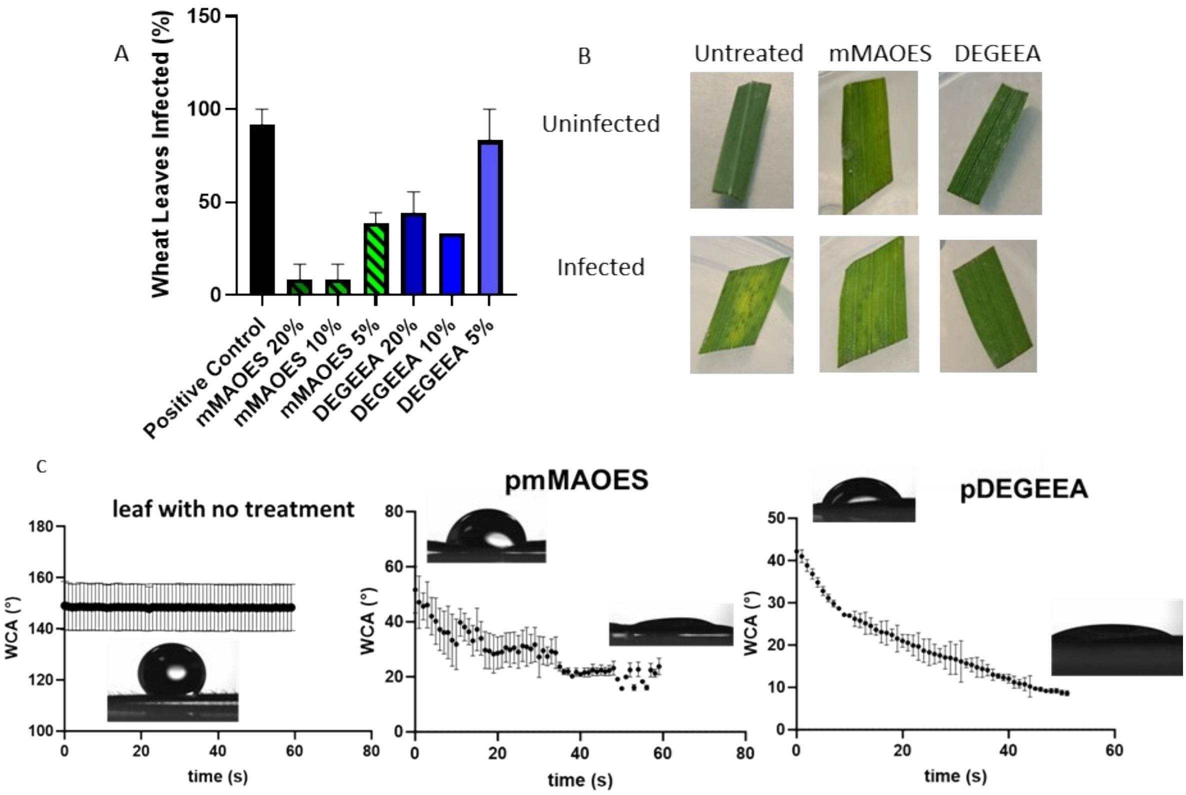

Figure S4. Protective effect of polymers against *B. cinerea* on wheat leaves. (A) Percentages of leaf segments scored visually as infected (±SETM, n=6) 3 days after inoculation with *B. cinerea.* Leaf segments were either untreated or sprayed with the indicated polymers at 20%, 10%, and 5% w/v (using 20% v/v of isopropanol solvent) and were assessed visually. (B) Example images of wheat leaf segments treated as in (A), here with or without pmMAOES or pDEGEEA and sprayed or not with *B. cinerea* spore suspensions. Infection lesions are evident as small dark patches (~20 lesions are visible on the untreated/infected panel). (C) WCA measurements of wheat leaf segments either untreated or treated with 10% (w/v) pmMAOES or pDEGEEA. Images of droplet profiles (examples shown) were taken for 60 s and over three different leaf areas for each polymer from which average and standard deviation values were calculated when leaf sample segments allowed

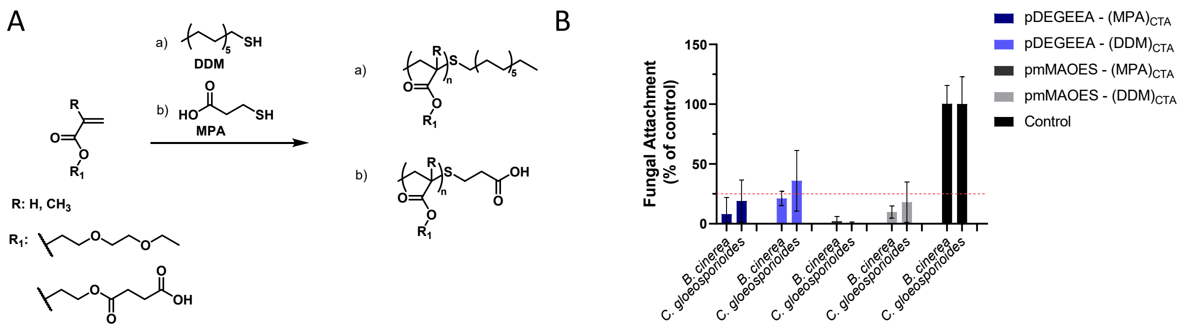

Figure S5 Comparison of MPA and DDM as chain transfer agents (CTAs) for scale-up polymerisations. (A) Scheme and structures for thiol-mediated free radical polymerisation of pDEGEEA and pmMAOES. (B) Anti-attachment activity of pDEGEEA and pmMAOES produced via either MPA or DDM as the CTA. Data show polymer attachment by spores of *B. cinerea* or *C. gloeosporioides* relative to the polystyrene control. The red dotted line signifies 25% attachment relative to the control (±SETM from three biological replicates).
